# Empowering Community Leadership: Perspectives of Peer Mentors Facilitating a Food Pantry-Based Nutrition Education Program

**DOI:** 10.3390/ijerph20032604

**Published:** 2023-01-31

**Authors:** Tracy L. Oliver, Lisa K. Diewald, Amy McKeever, Cerena A. George, Rebecca Shenkman

**Affiliations:** 1 M. Louise Fitzpatrick College of Nursing, Villanova University, Villanova, PA 19085, USA; 2 MacDonald Center for Obesity Prevention and Education, M. Louise Fitzpatrick College of Nursing, Villanova University, Villanova, PA 19085, USA

**Keywords:** peer mentors, food insecurity, food pantry, community health educators

## Abstract

Peer Mentors (PMs) are valuable health educators within food-insecure communities; however, little is known about PMs’ perspectives and experiences after serving in their peer mentor role. Therefore, this qualitative study explored PMs’ (n = 10) perceptions and analyzed data using thematic analysis based on descriptive phenomenology. Four themes emerged: (1) Successes and Struggles in Sharing Nutrition Knowledge; (2) Establishing a Conducive Learning Environment; (3) The Peer Mentor and Mentee Connection: Impact of Shared Experiences; (4) Empowerment of the Peer Mentor Experience. PMs have many advantages; however, more research is needed to evaluate the sustainability and efficacy of PMs within food-insecure communities.

## 1. Introduction

Recent estimates from 2021 indicate that 10.2% of Americans living in the United States were food insecure at least once during the last year, while 89.8% of U.S. households were food secure [1]. Food insecurity is defined as the state of being without reliable access to enough affordable and nutritious food and is an ongoing epidemic with social, educational, economic, and health outcome implications, whereas food security suggests there is access by all people to adequate food at all times [2]. Food insecurity specifically reduces the access, quality, variety, or desirability of one’s diet. Additionally, low-income and minority populations continue to experience greater food insecurity rates compared to other populations [3]. Taking it one step further, the concept of nutrition security builds on food security, emphasizing the co-existence of food insecurity and diet-related diseases and disparities and is among the United States Department of Agriculture’s (USDA) top priorities [4].

Food pantries (FPs) were initially designed as a supplemental food source, but presently are necessary for many families on an ongoing basis, to bridge the meal gap by providing supplemental food [5]. Many food pantries added health and nutrition education programs designed to encourage healthier food choices, inclusive of lessons on nutrition and meal planning for patrons, many of whom are plagued with chronic health conditions with a greater rate than the general population [6]. In studies of clients utilizing food pantry services, 24–34% self-reported a history of diabetes [7,8], and 44–72% reported hypertension [9,10]. Evidence supports improved food choices and health-promoting behaviors among patrons receiving nutrition education at food pantries [11,12,13]. Programs such as these inherently address the nutrition security of their clients. However, successful development of FP-based programs requires knowledge of the community’s values, traditions, and practices alongside an understanding of environmental and societal barriers that result from living with food insecurity. More specifically, parent-focused nutrition education programming should address health-promoting behaviors and food literacy skills, such as cooking self-efficacy, which teach proficiency in critical and functional food-related knowledge in order for parents to provide a healthy food environment for their families [14]. The modality of how best to deliver such programming is evolving.

Health promotion and nutrition education programming delivery by community members serving as peer mentors (PMs) is an innovative approach and documented strategy to reach low-income, minority, or socioeconomically disadvantaged populations [15,16,17,18]. PMs can be described as individuals who are trained and utilized to deliver a structure curriculum to a specific audience while providing guidance, social support and assistance [15]. Moredich and Kessler found that PMs who represent their community have an in-depth understanding of their community’s needs and can appropriately tailor the messages delivered [19]. In addition, they noted that community members perceive PMs as credible sources because they are respected members of that community, share similar cultural beliefs or health practices, and have experienced similar life challenges, which make them able to connect and relate to individuals on a personal level [15,16,17,18,19]. Baker and colleagues identified 28 best practices for effective nutrition education programs for low-income audiences under five domains, and relatability was identified as a key educator characteristic [20]. PMs have been successful when appropriately trained in nutrition to deliver health information or in facilitating changes in health-related behaviors and may strengthen social networks, potentially boosting motivation to make positive behavioral changes [21,22,23]. Research citing the peer mentoring model within an FP is still a relatively new addition to health-related education and community outreach literature. However, it holds promise by empowering and engaging food-insecure populations to take charge of their health through nutrition knowledge and food literacy development [17,18]. 

While using a PM model to facilitate health behavior changes and knowledge acquisition among community members has been established, PMs’ perceived benefits, feedback, and perspectives after delivering targeted programming, specifically within the FP setting, are yet to be fully established or explored. Using a focus group and interviews, this paper reports on perspectives, experiences, successes, and challenges of delivering an FP-based, parent-focused nutrition education program by a select group of PMs.

## 2. Materials and Methods

This qualitative study was employed to explore the experiences and perspectives of PMs after they completed teaching a nutrition education series to a parenting group within a FP.

### 2.1. Setting

Martha’s Choice Marketplace (MCM) is the largest FP in Montgomery County, a suburb in Southeastern Pennsylvania, and the site that supports this program [24]. MCM provides monthly food assistance to 1000 families (3500 individuals) but resides in the second most affluent county in the state, resulting in a high cost of living and a resultant income gap [24,25]. This region has approximately 19.5% of households living below the federal poverty line which is higher than the national average of 12.3% [26]. The PMs involved in this program were originally recruited as part of a previously described Community Cooks peer mentor-led nutrition education program that started in spring 2018 [17,18]. Since the program’s inception, Community Cooks’ PMs hosted three workshops that were open to the entire MCM community in addition to leading a four session series of parenting classes. It is this four session series of parenting classes, where PMs delivered a nutrition education curriculum to twelve parent participants, that preceded and is the basis of the reported focus group. The university Institutional Review Board (IRB) approved this study, IRB-FY2019-85 and PMs consented for the focus group conducted in 2019.

### 2.2. Participants

Ten of the eleven PMs from the original Community Cooks program consented to continue to serve in their PM role in a parenting skills class featuring healthy eating and basic cooking skills [17,18]. Prior to their participation in the parenting skills class, PMs were trained and evaluated by Registered Dietitian Nutritionists (RDNs) on (1) basic nutrition education and cooking skills, and (2) strategies for teaching simple nutrition education and healthy cooking skills to twelve participants in four consecutive one-hour sessions. Following the four session series, PMs were invited to participate in a post-program focus group and interview to share their experiences. PMs were compensated for participating in the session. Since the overall aim of this focus group was to assess the perspectives among PMs following the group program, the sample size was limited to PMs with this specific experience and insight. While smaller than other focus group studies of similar intent [27], this focus group was intentionally limited to these PMs because of the unique nature of their shared experiences, and therefore, focus group participation was not open for additional recruitment. PM demographics characteristics are reported elsewhere [17].

### 2.3. Procedure

MCM hosted the four-week PM-led nutrition education program within parenting classes. Up to three PMs rotated weekly to deliver a 60 min nutrition education lesson, including an interactive activity and healthy recipe preparation. In preparation for each week, the selected PMs practiced delivering the content and were encouraged to prepare the recipes at home.

### 2.4. Instruments

A semi-structured one-hour focus group was conducted with the PMs at the end of the program, and the session was audio recorded. The focus group included questions about the PM’s self-efficacy, confidence as a mentor, and any barriers to serving in this role. PMs were also asked to report on observations of the program’s effectiveness within this community and the participants’ receptiveness to the lesson content.

## 3. Data Analysis

The focus group recording was professionally transcribed, and two research team members independently reviewed the transcripts to correct any errors. Data were analyzed using Colaizzi’s phenomenological approach to identify emerging patterns [28]. Initial themes were identified for each guiding question. Next, repeating ideas were compared to ensure that each category adequately conveyed the participants’ perspectives. The process continued until all pertinent data were discussed and categorized. Finally, the summarized data were written in the narrative description and reviewed for clarity and accuracy. These findings were used to draw conclusions about the PMs’ perceptions of their PM role.

## 4. Results

The following four themes emerged: (1) Successes and Struggles in Sharing Nutrition Knowledge; (2) Establishing a Conducive Learning Environment; (3) The Peer Mentor and Mentee Connection: Impact of Shared Experiences; and (4) Empowerment of the Peer Mentor Experience. See Table 1 for list of themes and supportive quotes.

### 4.1. Theme 1: Successes and Struggles in Sharing Nutrition Knowledge

The PMs felt successful in their ability to share nutrition information and recipe preparation tips with the program participants and felt confident and prepared to deliver the nutrition lessons they had been trained on in preparation for the series. However, regardless of how well versed the PMs were in the lesson content, some participants were eagerly engaged while others were not as seemingly receptive. The PMs met with some resistance and had to overcome challenges presented by participants who were hesitant to try different foods and recipes or rejected specific messaging based on their personal or cultural preferences. The PMs observed the varied interest and enthusiasm levels of participants and some described participants wanting them to hurry up and finish the lesson or the recipe preparation so they could taste the meal. In other cases, PMs felt a general resistance to trying the “healthy” recipes or new ingredients served and found that participants were more willing to try healthier versions of recipes only when familiar with them. Despite the resistance to trying “healthy” versions of recipes, PMs felt encouraged when participants tasted the meal, and were pleasantly surprised.

### 4.2. Theme 2: Establishing a Conducive Learning Environment

PMs expressed frustration and challenges motivating and engaging some participants in an orderly setting. It was difficult to balance the roles of being friendly and of being someone in charge while maintaining a respectful classroom environment. Participants frequently engaged in side conversations or were visibly distracted, which presented unique challenges for the PMs. PMs had to navigate between establishing professional boundaries and a conducive learning environment and remaining approachable to fellow group members. In general, PMs felt frustrated with the lack of respect or attention from the program attendees and felt additional training could have better prepared the PMs for their role.

### 4.3. Theme 3: The Peer Mentor and Mentee Connection: Impact of Shared Experiences

Another important theme was the recognition among PMs that the value of their presence, encouragement, and the nutrition knowledge shared went beyond the classroom setting. PMs discussed how unique personal experiences enhanced the nutrition information from the lesson among the group members. The PMs cited several examples of discussions with or comments by participants that signified a real desire to improve their nutritional health.

### 4.4. Theme 4: Empowerment of the Peer Mentor Experience

Finally, and likely the most salient theme and profound takeaway from their entire PM experience was the personal empowerment and confidence stemming from their role as a PM. The PMs discussed the positive dynamic and synergy created among their peers, which increased their self-confidence as competent leaders and enhanced their relatability to participants. PMs developed a personal sense of purpose and belonging through supportive relationships and the cohesive PM community they formed. Additionally, the PMs reflected on the program design, including aspects such as presenting as a team on specific topics which helped establish camaraderie and provided additional support and empowerment by having fellow PMs presenting together. The PMs also described a sense of personal relatability with the participants. PMs believed that the participants felt more comfortable learning from their peers rather than the research team from outside their community. They also perceived that having a shared background, similar story, and race/ethnicity enhanced this relatability and acceptance.

Of additional importance, one PM described how peer-to-peer relatability enhanced the program. Some of the PMs were able to communicate with participants in different languages. For example, one PM was of Middle Eastern descent and could translate the lesson content and overcome language barriers to those participants who spoke Arabic. The value this PM brought to the group simply by translating information cannot be overlooked. Not only does this skill empower the PM, but it empowers the participant to properly comprehend the information.

## 5. Discussion

Food insecurity among American households remains one of the social and economic factors that the literature has linked with poorer health, increased rates of chronic disease, and higher healthcare costs [29]. Healthy People 2030 highlights the goal to improve food security and health by promoting healthy eating and making nutritious food available [30]. Within the context of this goal, it underscores the concept of nutrition security which emphasizes not only the need for increasing knowledge about making healthy food choices but also increasing the accessibility of these foods for all which ultimately could help prevent diet-related diseases and disparities [4,30]. Public health interventions must focus on utilizing more evidence-based nutrition education programs to meet these needs of the underserved. Previous research already shows that incorporation of a PM-led nutrition education programs in a FP setting may be a viable solution to reach underserved community members and provide resources related to healthy eating [18,30,31]. Therefore, the idea of using PMs to deliver nutrition education to communities of need at large may be a feasible and sustainable option to promote health within vulnerable groups [15,18]. The uniqueness of this research, however, is highlighting the intrinsic benefit that PMs get from doing meaningful work, performing it well, and being in a leadership position.

Very few published studies explore the perceptions of PMs after delivering a nutrition education program, none so within an FP setting. Typically, most PM research reports on the benefits to the recipients of the PM and community-based nutrition education, not the providers. The authors wanted to give a voice to the PMs so they could share and reflect upon their role and purpose as a PM. After delivering nutrition education sessions to an FP-based parenting group, the focus group questions were formulated to stimulate conversation regarding the PMs’ perspectives and provide overall program feedback. The themes reflect a variety of personal and group benefits in addition to challenges such as managing group dynamics and parent-participant engagement.

It should be highlighted that this focus group revealed that PMs were instrumental in providing a bridge for delivering simple nutrition messages and skill-building sessions to fellow FP clients and offered a valuable community-facing option for nutrition education program delivery. These findings align with PM literature in that PM-led programs are noted to have sustainable and long-term impacts on participants as well as can enhance health education efforts [15,16,21,22,23]. Empowering PMs within an FP community setting with the appropriate knowledge and skills to impart nutrition education to their peers can be an effective approach to building individual and community relationships that lead to positive health changes [15,16,18]. Benefits extend to both mentors and mentees, and outside experts do not easily accomplish this connection between the two groups [15,16]. The PMs vocalized how their relatability to participants fostered a trusting relationship, and the PMs felt empowered to serve as effective facilitators and share their knowledge with the group. However, despite their confidence and successes, the PMs also described the barriers faced when conducting the lessons, such as managing classroom social dynamics and some participants’ hesitancy to taste new foods or be open to the lesson content. Findings from this focus group demonstrate that the success of PM-led nutrition education programming may be improved through a leadership skills training program that includes attention devoted not only to content but to engagement and session management strategies as well.

### 5.1. Strengths and Limitations

This project explored PMs’ unique perspectives after delivering a PM-led nutrition education program within a community with a high prevalence of food insecurity. These results contribute to the body of research on improving nutritional health in low-income communities and can inform the design of future programs utilizing PMs or community health educators [15,16,21,22,23]. However, the findings of this study should be interpreted while considering the study’s limitations. PMs were recruited from within the MCM community as part of a previous peer mentor-led nutrition education program (Community Cooks) [17,18]. Additionally, the parents who participated in the nutrition education program were also recruited from a restricted geographic area served by the FP. The PM focus group sample size was small; therefore, this may limit the generalizability of these findings. Finally, although the PMs, as well as the parent participants, represented a diverse group in terms of race/ethnicity that often relies on federally funded food assistance programs, this study’s results were not considered an exhaustive exploration of all PM roles in delivering nutrition education to food-insecure populations.

### 5.2. Future Directions

Peer mentor health educators in a community setting like an FP can inspire individuals and communities to make better health choices. Peer mentoring is an effective strategy to bridge the gap between information dissemination and information retention, and community programming is necessary to reach people outside the traditional health care setting [15,16]. However, in addition to providing guidance on culturally relevant, economically feasible and simple messaging, inclusive PM training should include guidance on fostering participant engagement while establishing ground rules and expectations for behavior during sessions. These foundational skills will enable PMs to successfully encourage positive social engagement and promote supportive relationships to ensure a conducive learning and skills building environment.

## 6. Conclusions

Peer mentor-led nutrition education delivered in an FP setting targeting a low-income, food-insecure community can be considered an innovative and sustainable teaching approach. Additionally, based on PM qualitative feedback, the PMs may have also found the program to be an invaluable and empowering experience that may infuse benefits far beyond the program implementation, such as organizational skills, leadership, teaching, and public speaking for the PM participants. The PMs also offered tangible suggestions for training improvement that might best equip PMs for some of the challenges related to delivering educational programs in a group setting. Future research is needed to explore the sustainability efforts and potential health impacts and overall benefits on PMs, the patrons, community members, and parent participants, within the FP setting and beyond.

## Figures and Tables

**Table 1:** Emergent Themes with Illustrative Quotes.

Theme	Quotes Supporting Theme
Theme 1: Successes and Struggles in Sharing Nutrition Knowledge	“We were able to share some knowledge with them of the different things they can get out of the pantry and actually cook them for their families because a lot of times people go to the pantry, especially the vegetables or the fruits, they’re like what do I do with it? So we were able to say, you could do this or that. I think it actually helped some people.” “The initial [lesson] is like, oh no, I’m not going to try anything. But then, by the second [lesson], people were starting to relax a little bit. Like, okay, I’ll try it. I might not like it. I might not eat it, but I’m going to try it. And then, as it progressed—more people started to be like you know what everybody else is trying stuff and they liked it.” “What I thought was cool [was] the children in the room. There were two women, they really didn’t want to try anything, and they had all these excuses of why they couldn’t. But then, when they saw the children eating the chili and the brownies, they tried it.” “I think they took more to meals they were closer to understand like [when] we were talking about chili. They seemed to be much more willing. Okay, I’ll try the chili. I’ll try brownies because those are things they’re used to, whereas if you try with a zucchini that they’ve never had and it’s like no.” “People don’t like to hear anything about healthy.” “I agree with her because the butternut macaroni and cheese, we had a couple [participants] that were like butternut what? But once they tried it, they were like looking in the pan is there anymore left.”
Theme 2: Establishing a Conducive Learning Environment	“I liked least that there was a lot of sidebar conversations that we had a hard time taking control of. It wasn’t so much as the calling out of, oh well, I have this idea or that. It was more of on the side of what we were trying to explain. But yeah, so I even noticed with that it was certain individuals were side talking, and it was making it difficult for the other participants to hear what our PMs were trying to say.” “The chatter at the table. They just kept talking. As we were doing our thing [teaching], they just [talked] amongst themselves. Like Participant 1 said, they just chatter, chatter, chatter, chatter. So I don’t know if it [nutrition content] sunk in or not.” “I would say more like crowd control. Maybe if we went over some basic etiquette for lack of a better word. Because I think that’s what it was that I don’t think the people were trying to be rude. I think they just did not know. Maybe they just didn’t know.” “And it [the side conversation] wasn’t quietly either. It was almost on the line of disrespectful. I don’t to hear you. I’m just going to have my own little conversation, and I don’t care what you’re doing. But you can’t really say, hey, shut up.”
Theme 3: The Peer Mentor and Mentee Connection: Impact of Shared Experiences	“I think people definitely got something out (of) it. There were two women who afterward we spoke for like another 15–20 min because we connected on the fact [we] were both diabetic, and they were battling having their sugar under control, and I was battling the same thing. So we were able to sit there and talk back and forth the different ways that we could have the foods we like healthier but still mind the sugar and all. And I learned from them just like they learned from me.” “I think we actually made a difference. The one gentleman, he was all about chips and pizza and cookies, and we actually made a difference. He was like, I want the recipe for the mango salsa, and I’m definitely going to make the salad again. I think we made—even if it might have been just one meal a week, I think we did something in his life.”
Theme 4: Empowerment of the Peer Mentor Experience	“I guess more confidence because I had some oral issues that were very uncomfortable for me to speak to people. So I did get confidence.” “I think it really did help with the confidence. I think if you were up there by yourself, I I would still be confident, but maybe not as outspoken as with having the team there.” “I like the team format. I think that it helps. It’s like a support system, and I think that it actually helped get the information across better because you have somebody by your side. And it gives you a little bit more confidence than if you’re up there by yourself. I think it works better. You know you’re there for each other, at least two people.” “I prefer to have somebody with me because I don’t know everything. I forget things.” “We had the same thing. [When] we did the first one, and I was scared to death, so we went in and practiced. But then I saw I had two other people with me so I really can’t make a fool out of myself.” “My favorite part is just how we worked together. We didn’t practice with each other. It just kind of came natural.” “I think it was very important because you have to look at the different dynamics. You have single parents. You have older individuals, younger individuals, different ethnicities, and I think the fact that we’re a culmination of those different things I think it really helped the parenting to say, okay, I can relate to her. They could find someone they could relate to, whether it was age-related, ethnicity, single parent, or being on a budget.” “Okay, that person [the PM] I can relate to as opposed to you as a dietitian or you as a nurse. Oh, well, they’re high and mighty. You went to college and paid all this money, so of course, you know what you’re talking about. But for us to come across and make sense to them and be able to use language that they know and stuff that jive[s] with them I think they were more absorbent to it.” “She was in one specific section translating, and that group of people was there, and then we would wait. She translated pretty fast.”

## Data Availability

Not applicable.

## References

[1] Coleman-Jensen A., Rabbitt M.P., Hales L., Gregory C.A., Hashad R.N. (2022). Prevelance of Food Insecurity and very Low Food Insecurity 2001–21. U.S. Department of Agriculture Economic Research Service. https://www.ers.usda.gov/data-products/ag-and-food-statistics-charting-the-essentials/food-security-and-nutrition-assistance/#:~:text=In%202021%2C%2089.8%20percent%20of,from%2010.5%20percent%20in%202020.

[2] Holben D.H., Marshall M.B. (2017). Position of the Academy of Nutrition and Dietetics: Food Insecurity in the United States. J. Acad. Nutr. Diet..

[3] Morales D.X., Morales S.A., Beltran T.F. (2021). Racial/Ethnic Disparities in Household Food Insecurity During the COVID-19 Pandemic: A Nationally Representative Study. J. Racial Ethn. Health Disparities.

[4] Food and Nutrition Security, U.S. Department of Agriculture. https://www.usda.gov/nutrition-security.

[5] Cooksey-Stowers K., Martin K., Schwartz M. (2018). Client Preferences for Nutrition Interventions in Food Pantries. J. Hunger. Environ. Nutr..

[6] Hardison-Moody A., Bowen S., Bloom J.D., Sheldon M., Jones L., Leach B. (2015). Incorporating Nutrition Education Classes into Food Pantry Settings: Lessons Learned in Design and Implementation. J. Ext..

[7] Kaiser M., Cafer A. (2018). Understanding High Incidence of Severe Obesity and Very Low Food Security in Food Pantry Clients: Implications For Social Work. Soc. Work. Public Health.

[8] Martin K., Wu R., Wolff M., Colantonio A., Grady J. (2013). A Novel Food Pantry Program. Am. J. Prev. Med..

[9] Liu Y., Zhang Y., Remley D., Eicher-Miller H. (2019). Frequency of Food Pantry Use Is Associated with Diet Quality among Indiana Food Pantry Clients. J. Acad. Nutr. Diet..

[10] Perkett M., Robson S., Kripalu V., Wysota C., McGarry C., Weddle D., Papas M.A., Patterson F. (2016). Characterizing Cardiovascular Health and Evaluating a Low-Intensity Intervention to Promote Smoking Cessation in a Food-Assistance Population. J. Community Health.

[11] Evans S.H., Clarke P., Koprowski C. (2010). Information design to promote better nutrition among pantry clients: Four methods of formative evaluation. Public Health Nutr..

[12] Flynn M.M., Schiff A.R. (2010). Raising the bar on nutrition: A program to improve diet quality and food purchasing for food pantry clients. J. Acad. Nutr. Diet..

[13] Martin K., Wolff M., Callahan K., Schwartz M.B. (2018). Supporting Wellness in Pantries: Development of a Nutrition Stoplight System for Food Banks and Food Pantries. J. Acad. Nutr. Diet..

[14] Coto J., Pulgaron E.R., Graziano P.A., Bagner D.M., Villa M., Malik J.A., Delamater A.M. (2019). Parents as Role Models: Associations Between Parent and Young Children’s Weight, Dietary Intake, and Physical Activity in a Minority Sample. Matern. Child Health J..

[15] Petosa R.L., Smith L.H. (2014). Peer Mentoring for Health Behavior Change: A Systematic Review. Am. J. Health Educ..

[16] Webel A.R., Okonsky J., Trompeta J., Holzemer W.L. (2010). A Systematic Review of the Effectiveness of Peer-Based Interventions on Health-Related Behaviors in Adults. Am. J. Public Health.

[17] Oliver T.L., McKeever A., Shenkman R., Diewald L.K. (2019). Barriers to Healthy Eating in a Community that Relies on an Emergency Food Pantry. J. Nutr. Educ. Behav..

[18] Oliver T.L., McKeever A., Shenkman R., Diewald L. (2020). Successes and Challenges of Implementing a Peer Mentor Model in an Emergency Food Pantry: A Qualitative Report. BMC-Nutr..

[19] Moredich C.A., Kessler T.A. (2014). Physical activity and nutritional weight loss interventions in obese, low-income women: An integrative review. J. Midwifery Women’s Health.

[20] Baker S., Auld G., Ammerman A., Lohse B., Serrano E., Wardlaw M. (2020). Identification of a Framework for Best Practices in Nutrition Education for Low-Income Audiences. J. Nutr. Educ. Behav..

[21] Smith L.H., Petosa R.L. (2016). A structured peer-mentoring method for physical activity behavior change among adolescents. J. Sch. Nurs..

[22] Smith L.H. (2011). Cross-age peer mentoring approach to impact the health outcomes of children and families. J. Spec. Pediatr. Nurs..

[23] Smith L., Petosa R., Shoben A. (2018). Peer mentor versus teacher delivery of a physical activity program on the effects of BMI and daily activity: Protocol of a school-based group randomized controlled trial in Appalachia. BMC Public Health.

[24] Martha’s Choice Marketplace Martha’s Choice Marketplace. http://marthaschoicemarketplace.com/.

[25] Most Affluent Counties in Pennsylvania. https://www.lehighvalleylive.com/news/2018/07/ranking_the_35_most_affluent_p.html.

[26] Data USA, Norristown. https://datausa.io/profile/geo/norristown-pa.

[27] Hoebeke R. (2008). Low-income women’s perceived barriers to physical activity: Focus group results. Appl. Nurs. Res..

[28] Colaizzi P.F., Valle R.S., King M. (1978). Psychological research as the phenomenologist views it. Existential Phenomenological Alternatives for Psychology.

[29] Özdemir D., Özdemir H.Ö. (2019). Health Care Cost of Socioeconomic Inequalities: A Pioneering Population-Wide Study. Am. J. Public Health.

[30] Office of Disease Prevention and Health Promotion Nutrition and Healthy Eating. Healthy People 2030. U.S. Department of Health and Human Services. https://health.gov/healthypeople/objectives-and-data/browse-objectives/diabetes.

[31] U.S. Department of Health and Human Services and U.S. Department of Agriculture (2015). 2015–2020 Dietary Guidelines for Americans. https://health.gov/our-work/food-nutrition/2015-2020-dietary-guidelines/guidelines/.

